# Correction to: Dynamic linear models guide design and analysis of microbiota studies within artificial human guts

**DOI:** 10.1186/s40168-018-0601-6

**Published:** 2018-11-27

**Authors:** Justin D. Silverman, Heather K. Durand, Rachael J. Bloom, Sayan Mukherjee, Lawrence A. David

**Affiliations:** 1 Program in Computational Biology and Bioinformatics, Duke University, CIEMAS, Room 2171, 101 Science Drive, Box 3382, Durham, NC 27708 USA; 2 Medical Scientist Training Program, Duke University, Durham, NC 27708 USA; 3 Center for Genomic and Computational Biology, Duke University, Durham, NC 27708 USA; 4 University Program in Genetics and Genomics, Duke University, Durham, NC 27708 USA; 5 Department of Molecular Genetics and Microbiology, Duke University, Durham, NC 27708 USA; 6 Departments of Statistical Science, Mathematics, Computer Science, Biostatistics & Bioinformatics, Duke University, Durham, NC 27708 USA

## Correction

Following publication of the original article [1], the authors noticed an error in the presentation of equations in the PDF version.

On page 10:

Equation 3 reads as:

$$ {\eta}_k={F}_k^{{\theta^{\hbox{'}}}_k+{\nu}_k,\kern0.5em {\nu}_k\sim N\left(0,\kern0.5em {V}_k\right)} $$

but should read as:

3 $$ {\eta}_k={F}_k^{\hbox{'}}{\theta}_k+{\nu}_k,\kern0.5em {\upsilon}_k\sim N\left(0,{V}_k\right) $$

On page 12:

The equation reads as:

$$ {\eta}_k={\left[{I}_R\otimes F\right.}_k^{{}^{\hbox{'}}\Big]{\theta}_k+{\nu}_k,\kern0.5em {\nu}_k\sim N\left(0,\left[{I}_R\otimes \kern0.5em {V}_k\right]\right)} $$

but should read as:

$$ {\displaystyle \begin{array}{l}{\eta}_k=\left[{I}_R\otimes {F}_k^{\hbox{'}}\right]{\theta}_k+{\nu}_k,\kern0.5em {\nu}_k\sim N\left(0,\left[{I}_R\otimes \kern0.5em {V}_k\right]\right)\\ {}\end{array}} $$

The original article has been updated.
