# Supplementary figures and images for: Dynamic linear models guide design and analysis of microbiota studies within artificial human guts
Source: Microbiome. 2018 Nov 12;6:202. doi: 10.1186/s40168-018-0584-3 (PMC6233358; doi:10.1186/s40168-018-0584-3)

Log Proportions

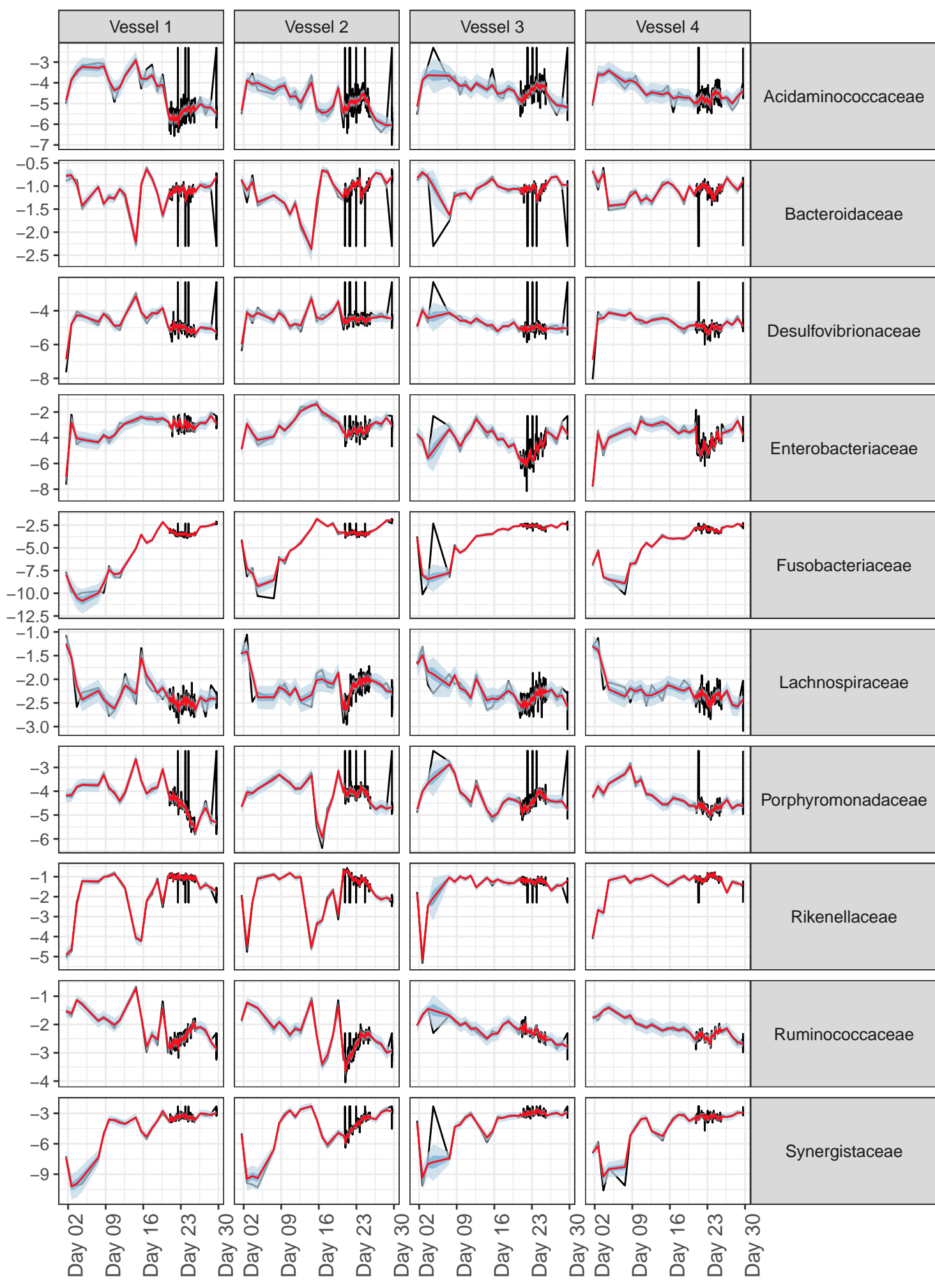

Supplement: Supplementary file 1 — Model fits to the observed data. Posterior mean (red), 50% (dark blue) and 95% credible (light blue) intervals for θt in terms of log transformed proportions (black). The raw count data are shown in black for comparison. A pseudo-count of 0.65 was added to the raw data prior to normalization and log-transformation to avoid taking the log of zero values. (PDF 163 kb) [file 40168_2018_584_MOESM1_ESM.pdf]

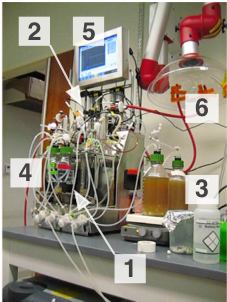

Supplement: Supplementary file 2 — Artificial gut setup. (1) Reactor vessels; (2) flow meters controlling gas inputs; (3) pump-fed media; (4) acid and base to regulate pH; (5) central controller; (6) snorkel for exhaust. Note that only two of four replicate vessels are illustrated in this photograph. (PNG 151 kb) [file 40168_2018_584_MOESM2_ESM.png]

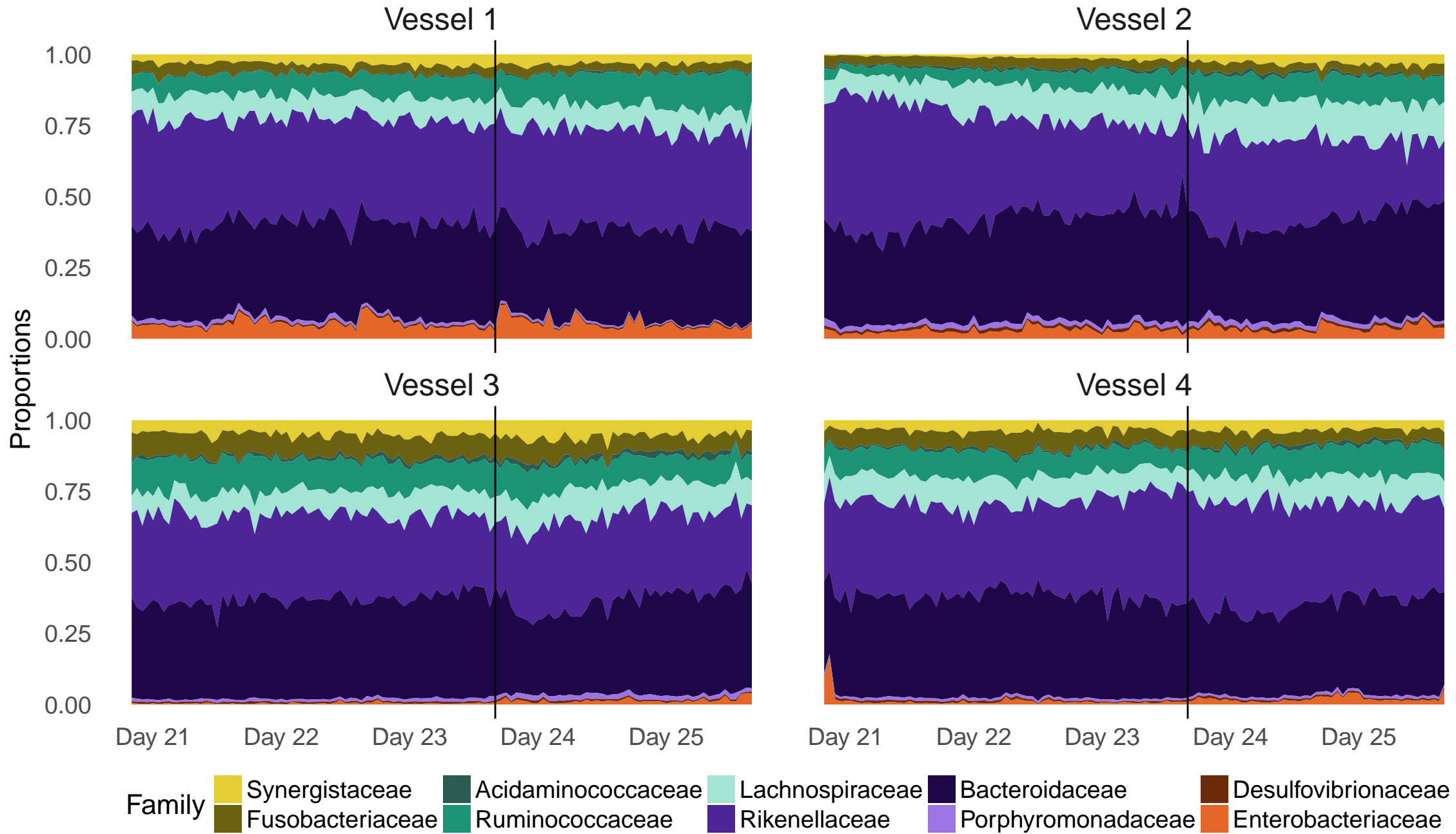

Supplement: Supplementary file 3 — Proportions of most abundant bacterial families estimated from count data during hourly sampling period. Proportions were estimated by dividing observed counts by the total number of counts observed for each sample. The time-point corresponding to B. ovatus supplementation is depicted as a black line. (PDF 37 kb) [file 40168_2018_584_MOESM3_ESM.pdf]

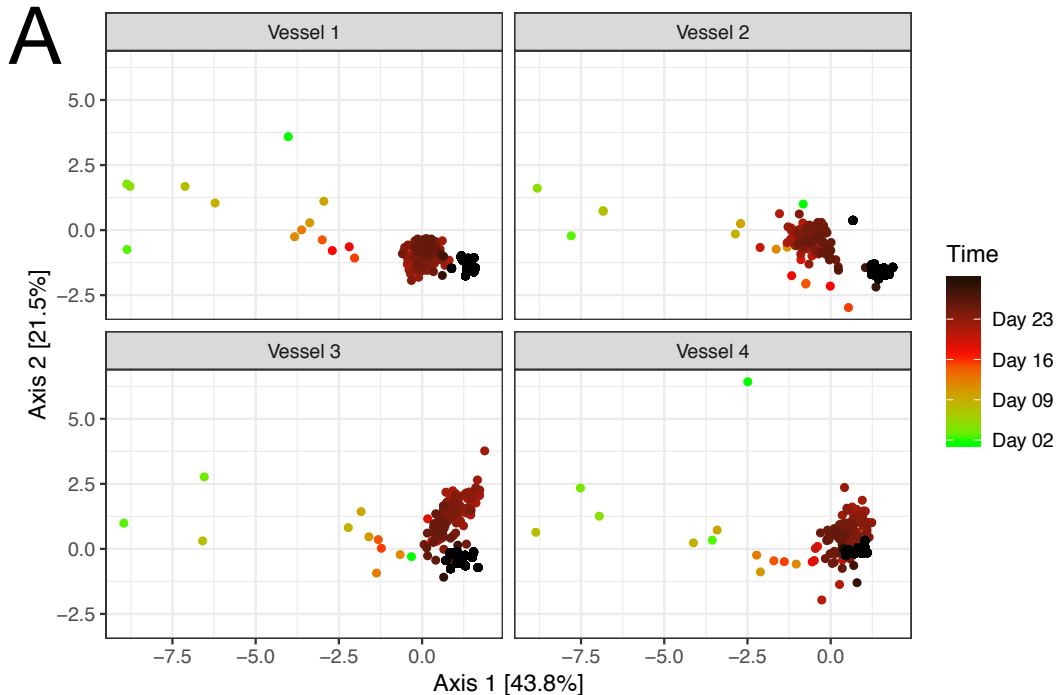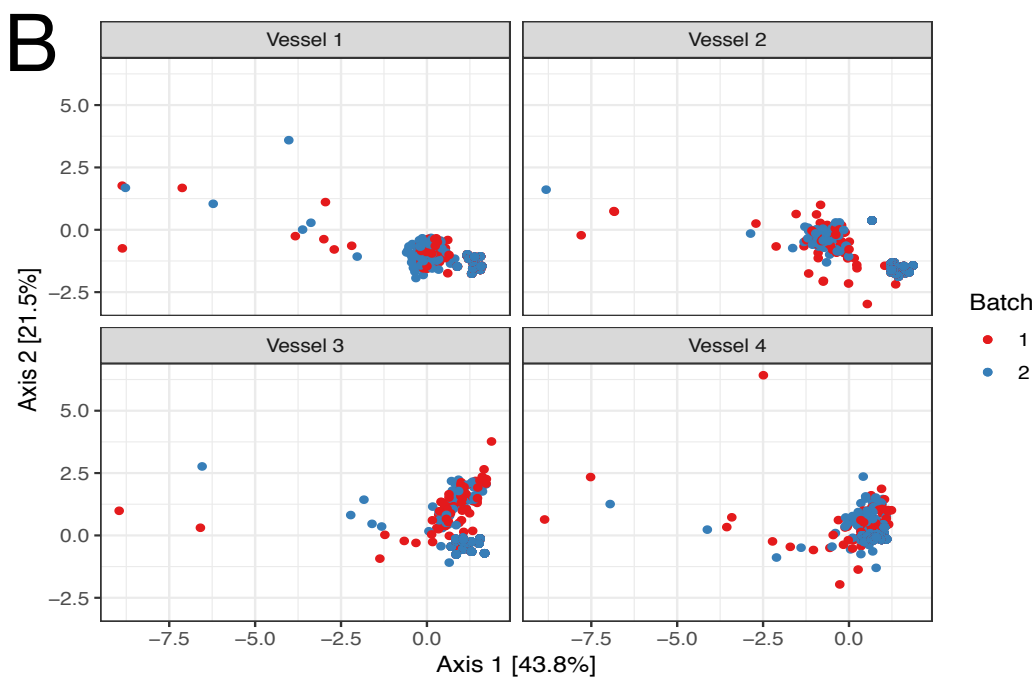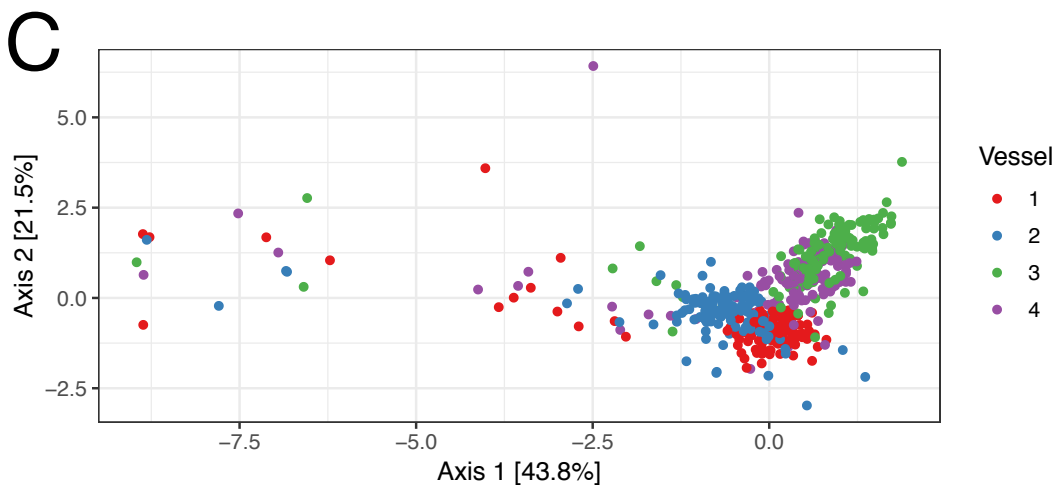

Supplement: Supplementary file 5 — PCoA based on Aitchison distance applied to most abundant bacterial families. To avoid taking the log or ratio of zero counts, a pseudo-count of 0.65 was added to all counts prior to calculation of the Aitchison distance. In panel (A) samples are labeled by collection time since the start of the experiment with technical replicates labeled in black. In panel (B) samples are labeled by sequencing batch and show no clear separation between batches. In panel (C) samples are labeled by artificial gut vessel. All results in Panels A, B, and C are shown with respect to the same two principle coordinates and are thus directly comparable across plots and panels. (PDF 87 kb) [file 40168_2018_584_MOESM5_ESM.pdf]

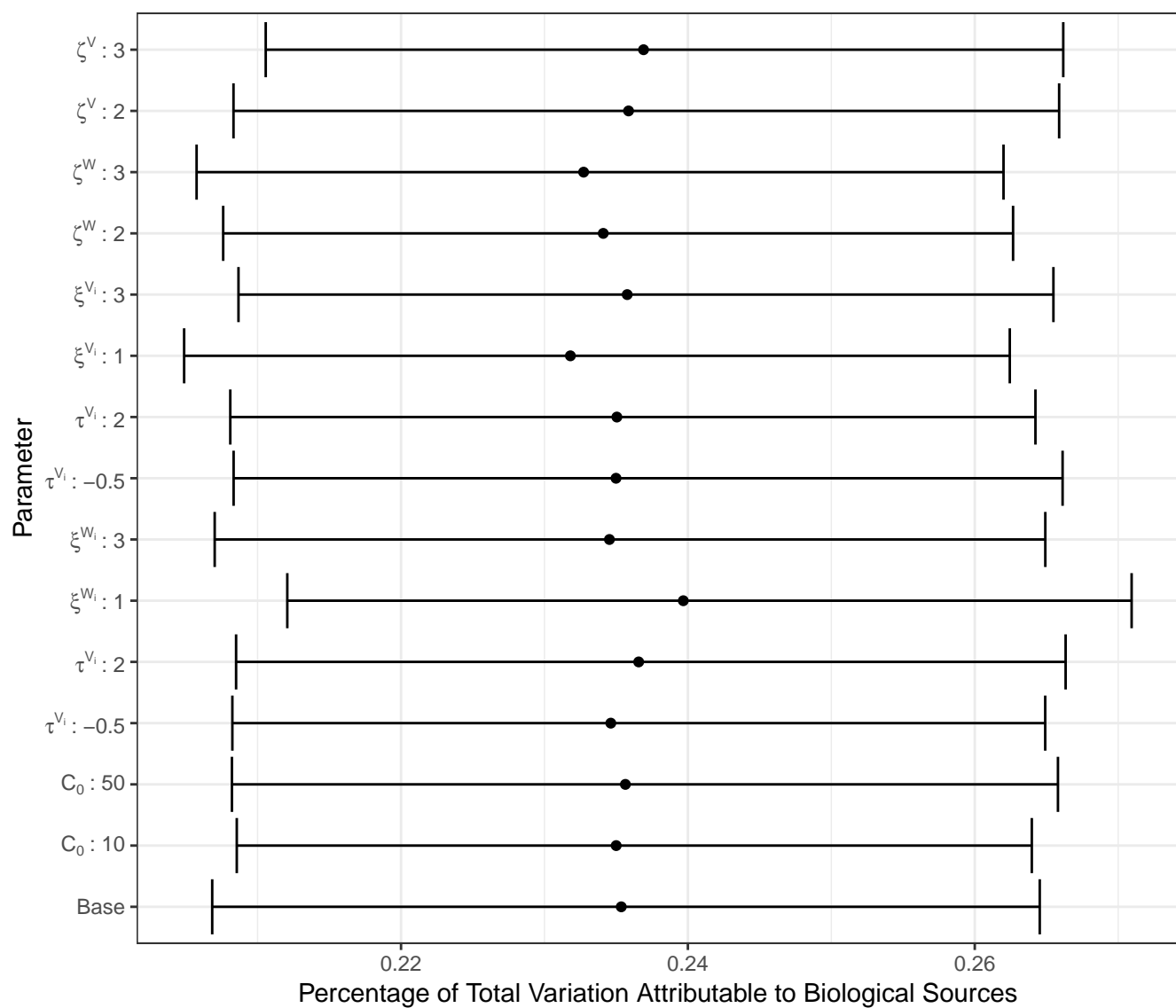

Supplement: Supplementary file 6 — Posterior estimates for the percent of total variation attributable to biological sources is not sensitive to modification of prior parameters. The “Base” prior parameter values refer to the values specified throughout the “Methods” section. In addition, the complete model was rerun with 14 separate prior parameters settings, each deviating from the Base values with respect to one parameter. Posterior 95% credible intervals and mean are shown for each set of prior parameters. (PDF 5 kb) [file 40168_2018_584_MOESM6_ESM.pdf]

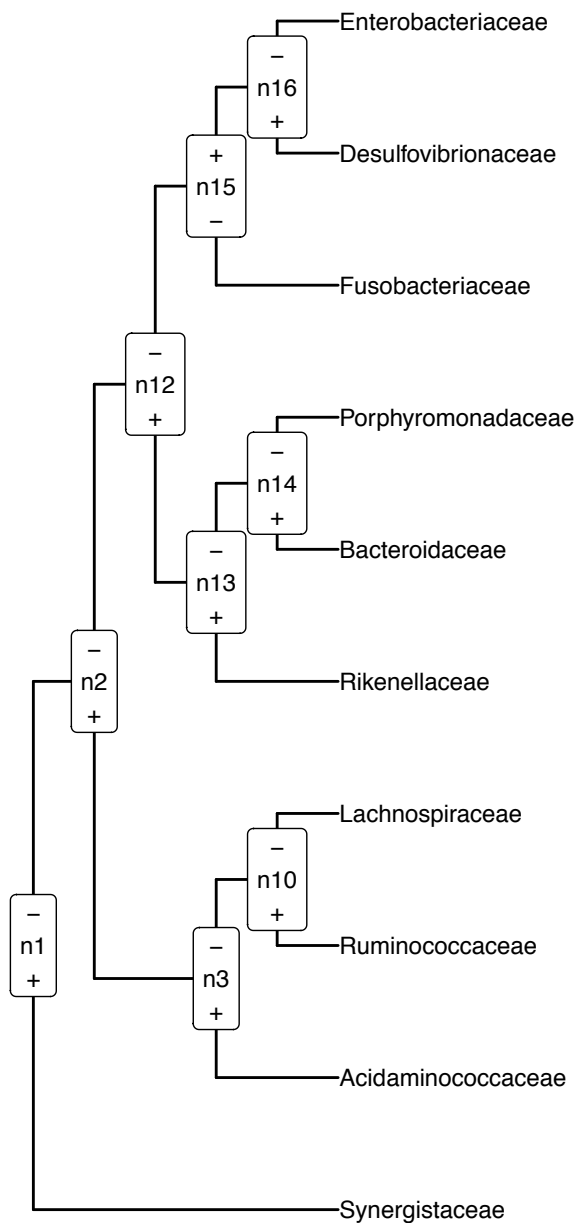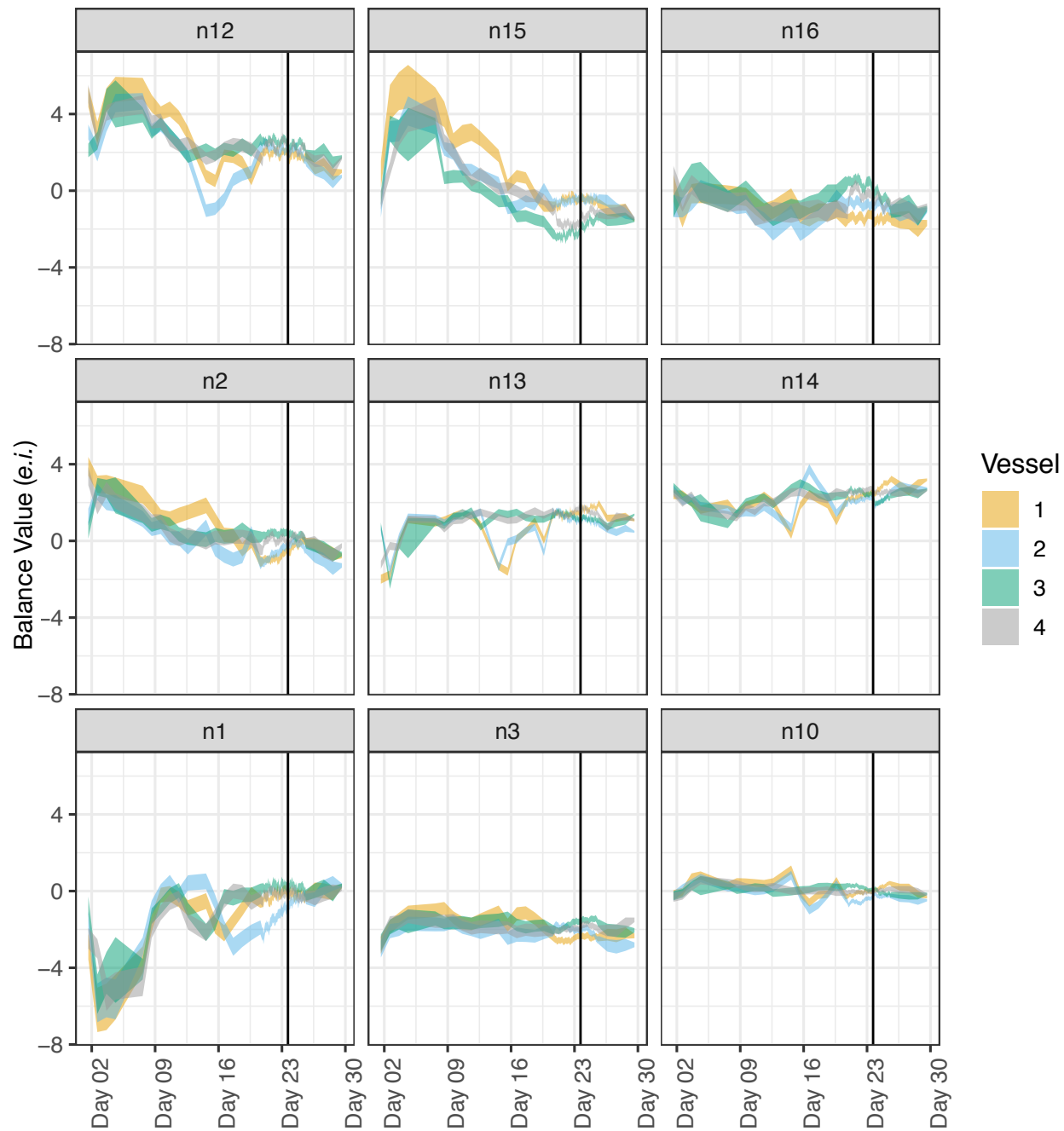

Supplement: Supplementary file 7 — Posterior 95% credible regions for bacterial dynamics (θ) in the PhILR Basis. (Left) The hierarchical tree of phylogenetic relationships between the bacterial families with PhILR balances (n1-n16, non-consecutive numbering) depicted (“Methods” section). Balance n12 is highlighted in Fig. 4. Branch lengths are not to scale. (+) and (−) refer to which subclade is found in the numerator or denominator of the balance respectively. (Right) Posterior 95% credible regions for the bacterial dynamics for each PhILR balance is depicted. The time-point corresponding to B. ovatus supplementation is depicted as a black line. (PDF 100 kb) [file 40168_2018_584_MOESM7_ESM.pdf]

Balance Value (e.i.)

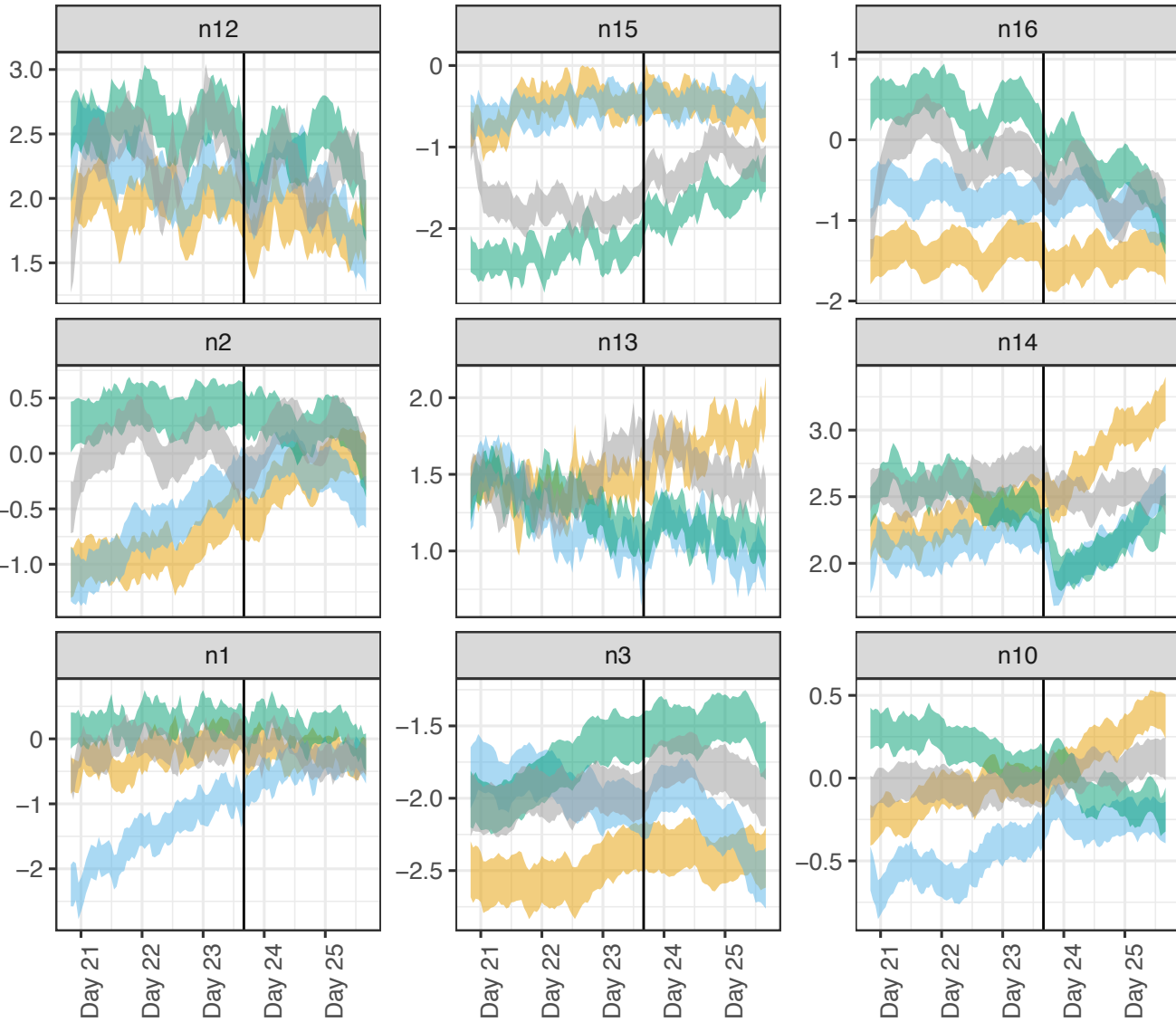

Supplement: Supplementary file 8 — Posterior 95% credible regions for bacterial dynamics (θ) for hourly samples in the PhILR Basis. Balances are defined in the left panel of Additional file 7, here highlighting the dynamics observed during the hourly sampling period. Balance n12 is highlighted in Fig. 4. Balance n14 shows a decrease in the ratio of the families Bacteroidaceae to Porphyromonadaceae. It is likely that this effect was due to the effects of fresh delivery media as balance shifts appear strongest in the control vessels that received sham treatment (media alone) (#2 and #3) compared to the treatment vessels that received media and B. ovatus (#1 and #4). (PDF 77 kb) [file 40168_2018_584_MOESM8_ESM.pdf]

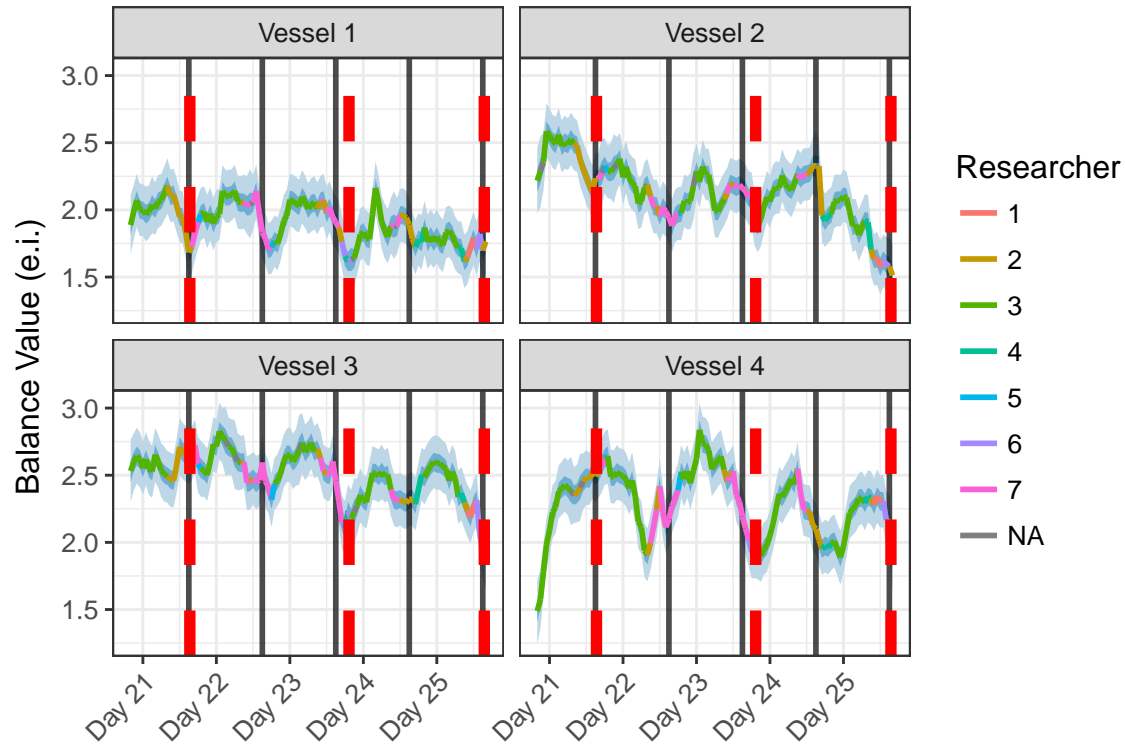

Supplement: Supplementary file 9 — Irregular sub-daily oscillation observed in PhILR balance n12 does not correlate with known external factors. As in Fig. 4b, the posterior mean and 95% credible interval of the microbial dynamics (θ) for balance n12 is shown during hourly sampling. The posterior mean is colored with the ID of the researcher who obtained each corresponding sample. Samples that were dropped from analysis due to low sequencing depth are denoted by NA for researcher ID. Times at which media feed bottles were changed are indicated with red dashed lines. Time-points corresponding to the daily sampling regimen are indicated by dark gray lines. (PDF 20 kb) [file 40168_2018_584_MOESM9_ESM.pdf]

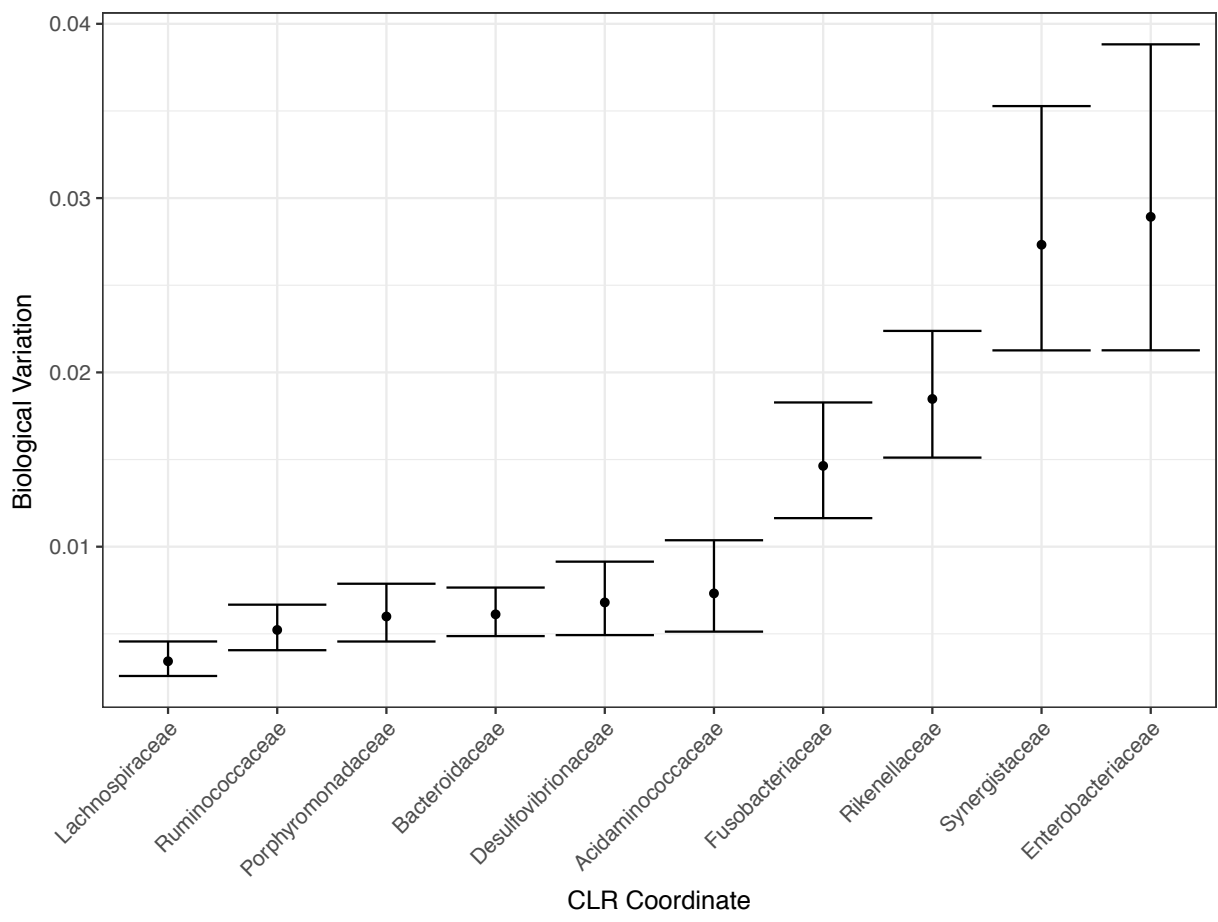

Supplement: Supplementary file 10 — Four bacterial families comprise most the biological variation in our study. Relative biological variation for each family was taken as the corresponding diagonal entry of the CLR transformed Biological variation matrix (WCLR; “Methods” section). The median and 95% posterior credible interval are depicted for each bacterial family. (PDF 20 kb) [file 40168_2018_584_MOESM10_ESM.pdf]

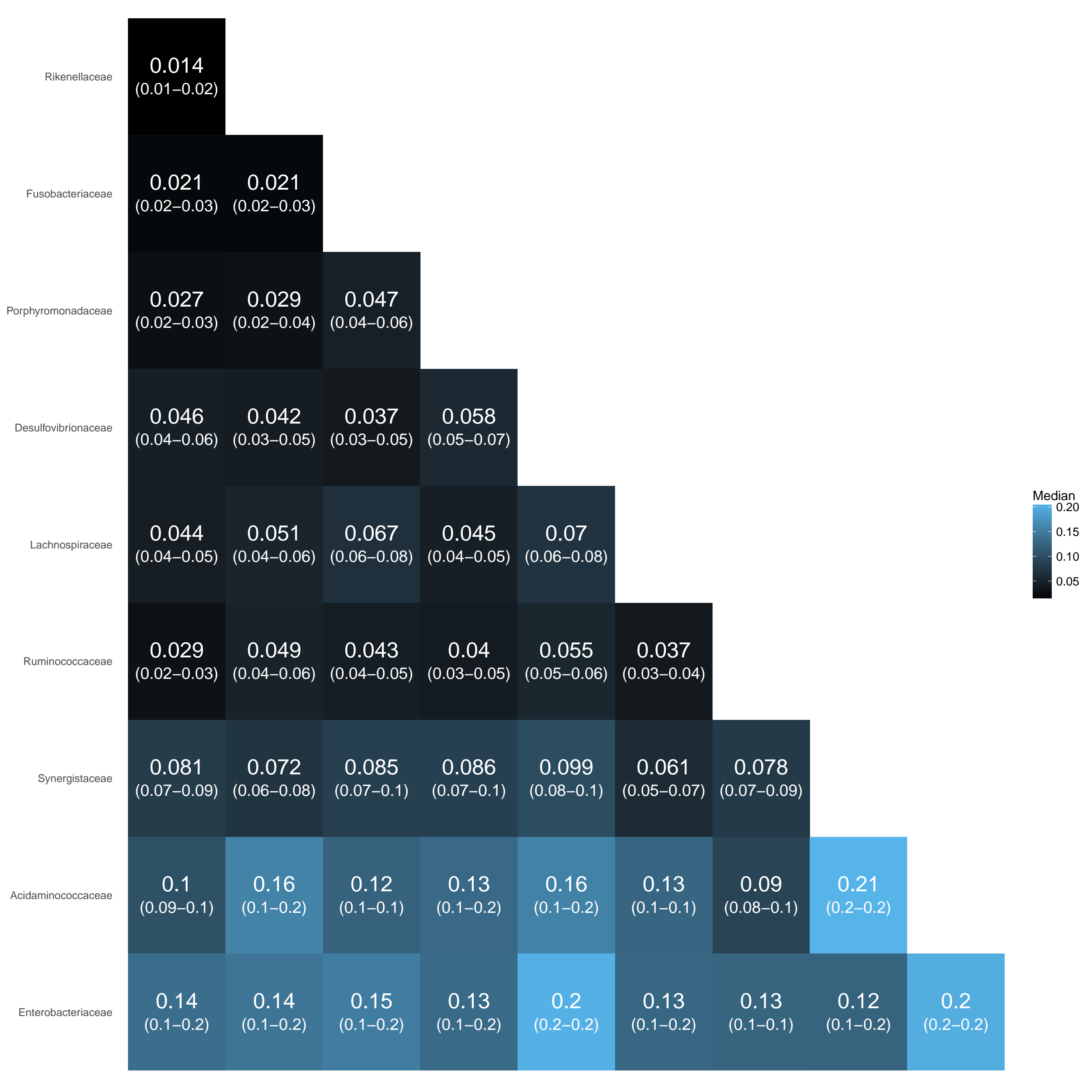

Supplement: Supplementary file 11 — The decomposition of technical variation among bacterial families. Posterior distribution for log-ratio variance (ρ) between pairs of bacterial families for technical variation (V). Heatmap color is given by the median of the posterior distribution of ρ. Each cell also gives the median and 95% credible region for the log-ratio variance (ρ) for the corresponding bacterial families. Columns and rows refer to the bacteria in the numerator and denominator of the corresponding log-ratios respectively. (PDF 5 kb) [file 40168_2018_584_MOESM11_ESM.pdf]

CLR Transformed Biological Variation

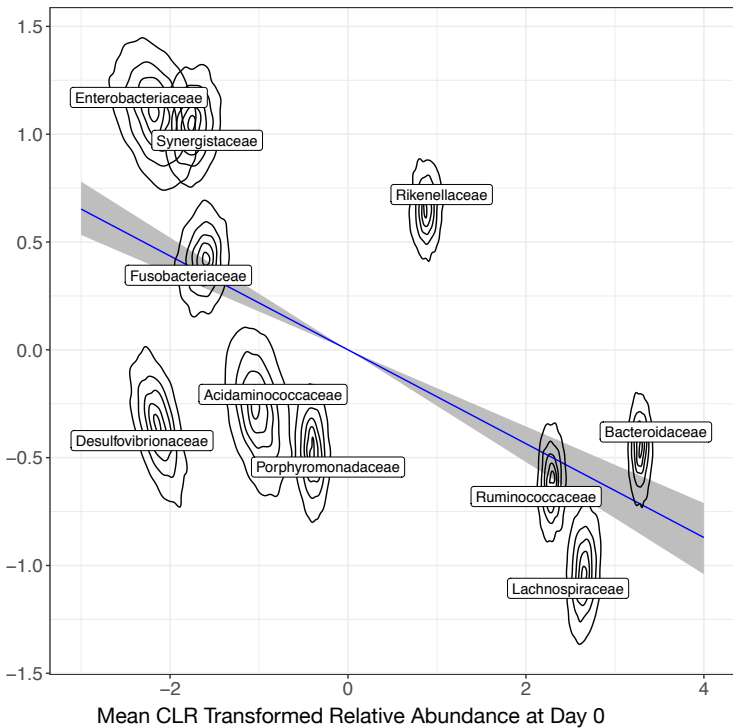

Supplement: Supplementary file 12 — An inverse relationship between biological variation and initial relative abundance. 5, 25, 50, 75, and 95% highest posterior density regions of the posterior distribution of mean relative abundances on day 0 and biological variation of the ten most abundant bacterial families. Both axes are CLR-transformed. Posterior mean and 95% credible regions are also shown for the regression between these variables (“Methods” section). (PDF 84 kb) [file 40168_2018_584_MOESM12_ESM.pdf]

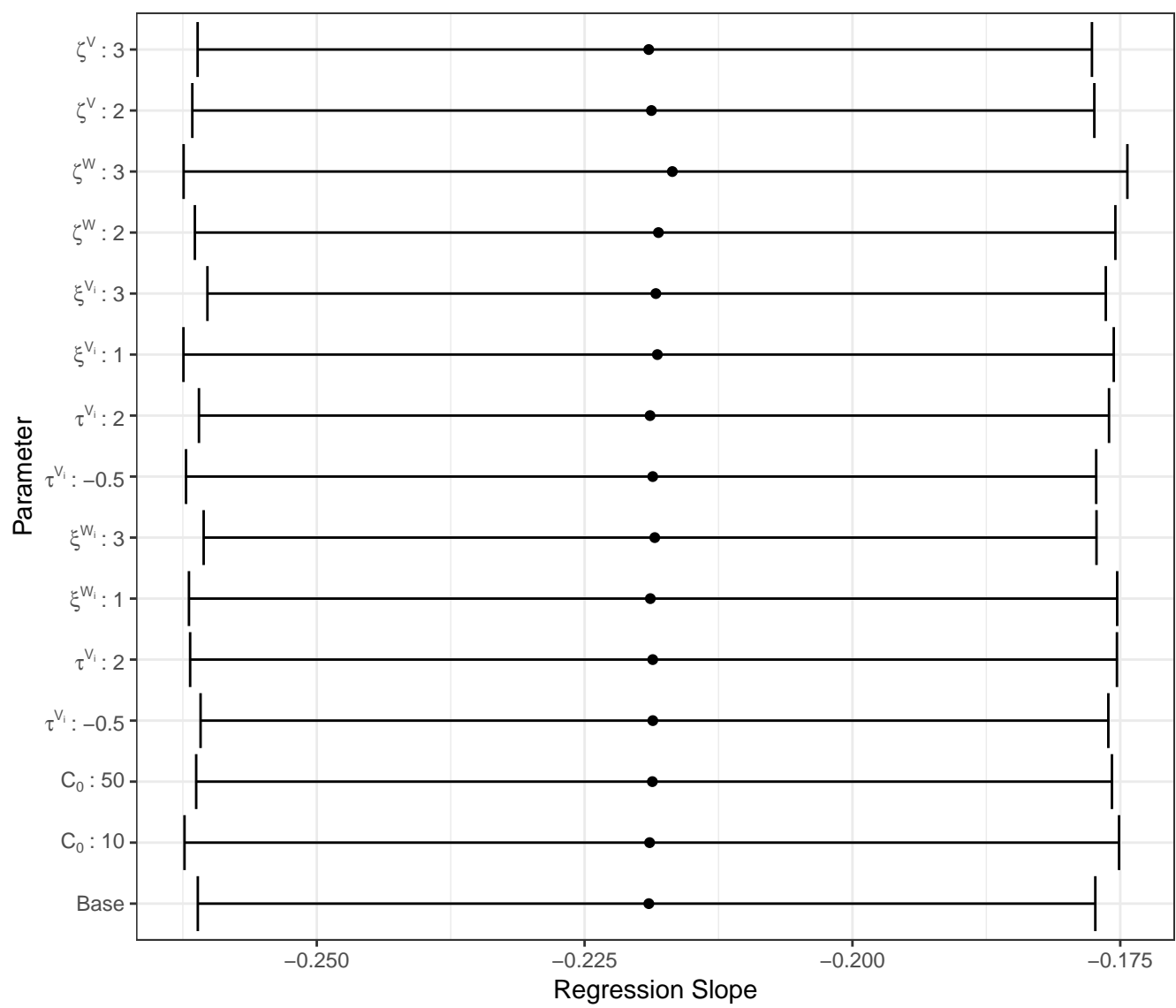

Supplement: Supplementary file 13 — Posterior estimates for the regression slope between biological variation and bacterial family starting relative abundance is not sensitive to modification of prior parameters. The “Base” prior parameter values refer to the values specified throughout the “Methods” section. In addition, the complete model was rerun with 14 separate prior parameters settings, each deviating from the Base values with respect to one parameter. Posterior 95% credible intervals and mean are shown for each set of prior parameters. (PDF 5 kb) [file 40168_2018_584_MOESM13_ESM.pdf]

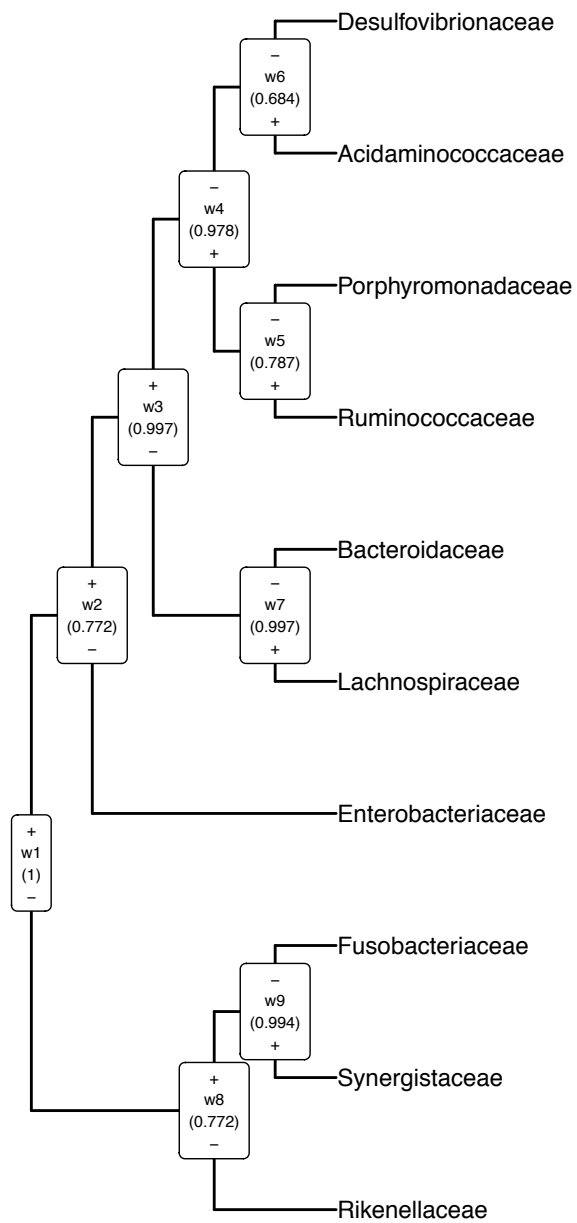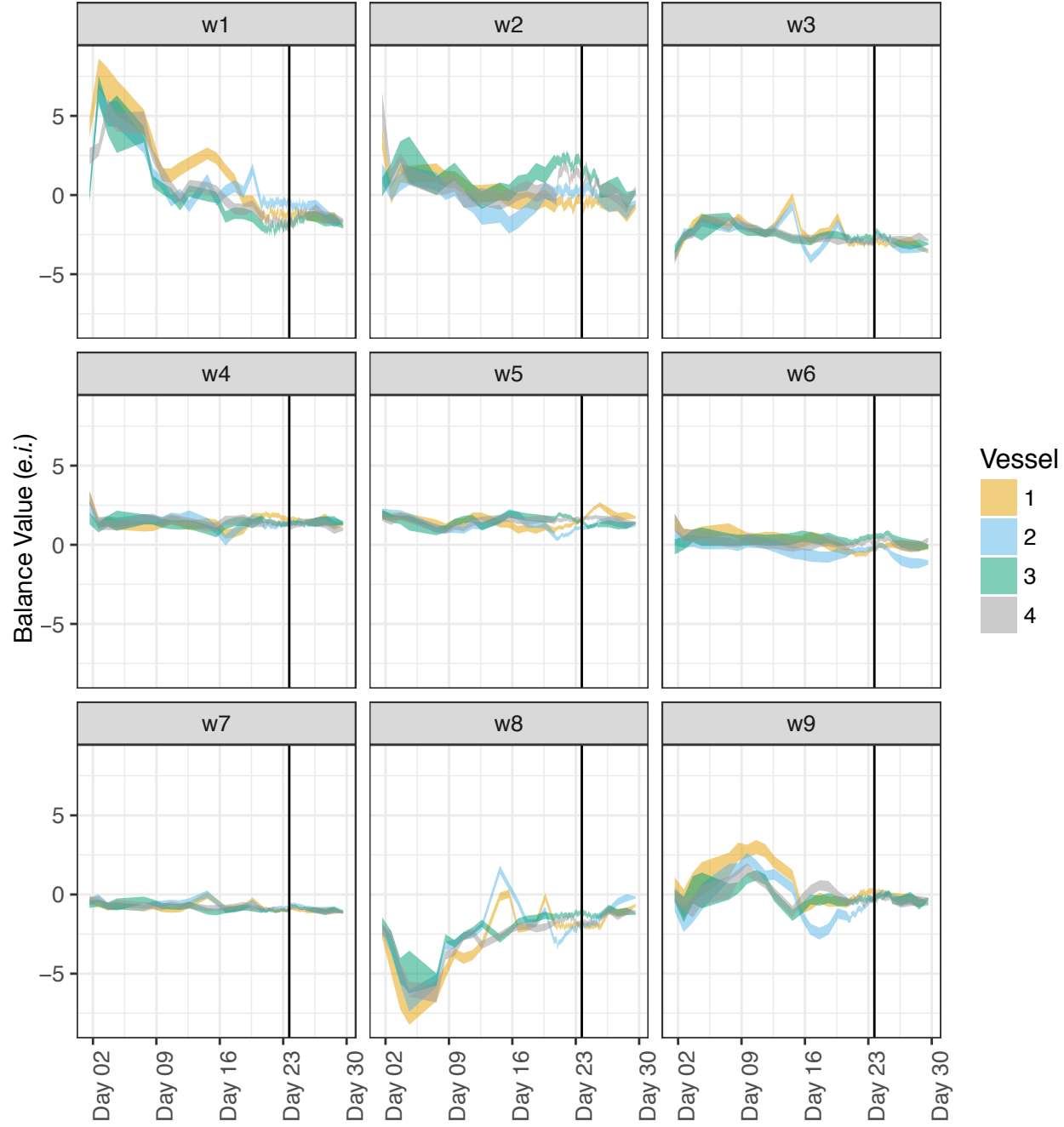

Supplement: Supplementary file 14 — Posterior 95% credible regions for bacterial dynamics (θ) in the Ward Basis. (Left) The consensus tree created by Ward clustering of bacterial families with Ward balances (w1-w9) is depicted (“Methods” section). Balances nearer the root of the tree display higher variance than balances nearer the tips (“Methods” section). (+) and (−) refer to which subgroup is found in the numerator or denominator of each Ward balance respectively. The proportion of samples from the posterior distribution in which a given bipartition was present is denoted under the corresponding balance name (“Methods” section). (Right) Posterior 95% credible regions for the bacterial dynamics for each Ward balance is depicted. The time-point corresponding to B. ovatus treatment is depicted as a black line. (PDF 89 kb) [file 40168_2018_584_MOESM14_ESM.pdf]

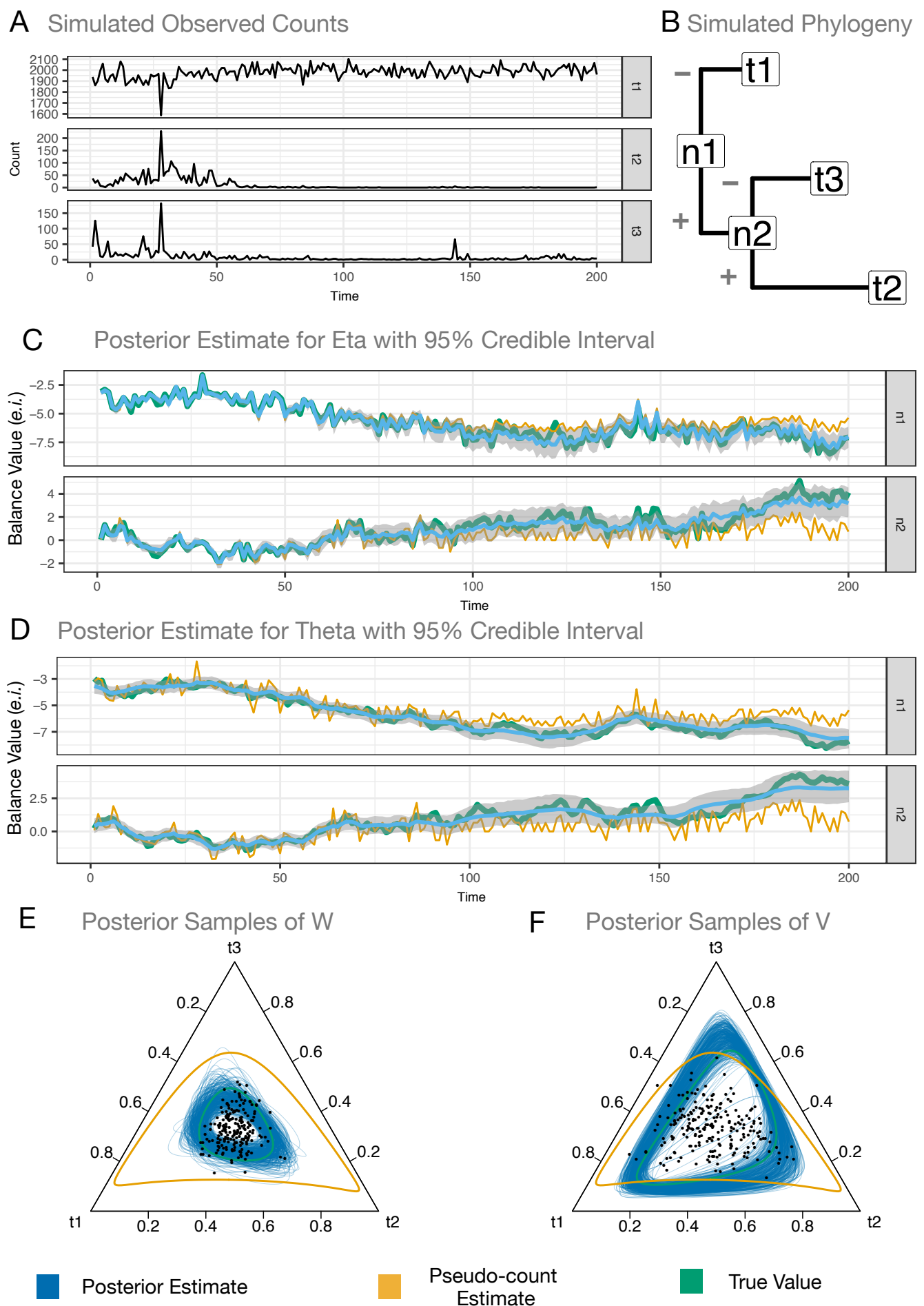

Supplement: Supplementary file 18 — Analysis of a toy simulated microbial community demonstrates the advantages of accounting for technical noise and uncertainty due to counting. (A) A 3 taxon (t1, t2, t3) microbial community was simulated according to the likelihood model used to analyze the artificial gut dataset (“Methods” section). Data from time-points 15, 16, and 20 were removed to simulate the effects of missing data on inferences. (B) A simulated phylogeny with annotated PhILR balances (n1, n2) used to analyze the simulated dataset. (+) and (−) refers to taxa in the numerator and denominator of associated balances. Pseudo-count based (PC) estimates for the multinomial parameters are obtained by adding 0.65 to all counts and then dividing each count by the sequencing depth of its associated sample and are shown as reference in (C-D). (C) Posterior mean and 95% credible interval for the multinomial parameters η. (D) Posterior mean and 95% credible interval for the unobserved microbial dynamics θ. PC estimates for the covariance of the multinomial parameters was obtained as the covariance of the first difference of the PC parameter estimates and are shown as reference in (E-F). 100 samples from the posterior distribution of the biological variation (W, E) and technical variation (V, F) depicted as 95% probability regions of the Logistic Normal distribution centered at the point in the simplex where each bacterial taxon is equally abundant. Black points represent the simulated biological variations (wt). (PDF 9820 kb) [file 40168_2018_584_MOESM18_ESM.pdf]
