# Supplementary material for: Dynamic linear models guide design and analysis of microbiota studies within artificial human guts
Source: Microbiome. 2018 Nov 12;6:202. doi: 10.1186/s40168-018-0584-3 (PMC6233358; doi:10.1186/s40168-018-0584-3)
Supplement: Supplementary file 4 — Samples were collected over a one month period with both daily and hourly sampling intervals. Daily samples were collected at 15:00 ± 00:30 h, hourly samples were collected within ± 10 min of depicted time. Samples that were either not collected or filtered from analysis due to low sequencing depth are shown in white, samples that were included in analyses are shown in blue. Samples from days 5, 6, and 13 were not collected due to holiday. In addition to standard hourly and daily longitudinal sampling, 20 samples were collected from the final time-point of each replicate vessel. The number of replicate samples that were included in the analysis are depicted in blue. (PDF 30 kb) [file 40168_2018_584_MOESM4_ESM.pdf]

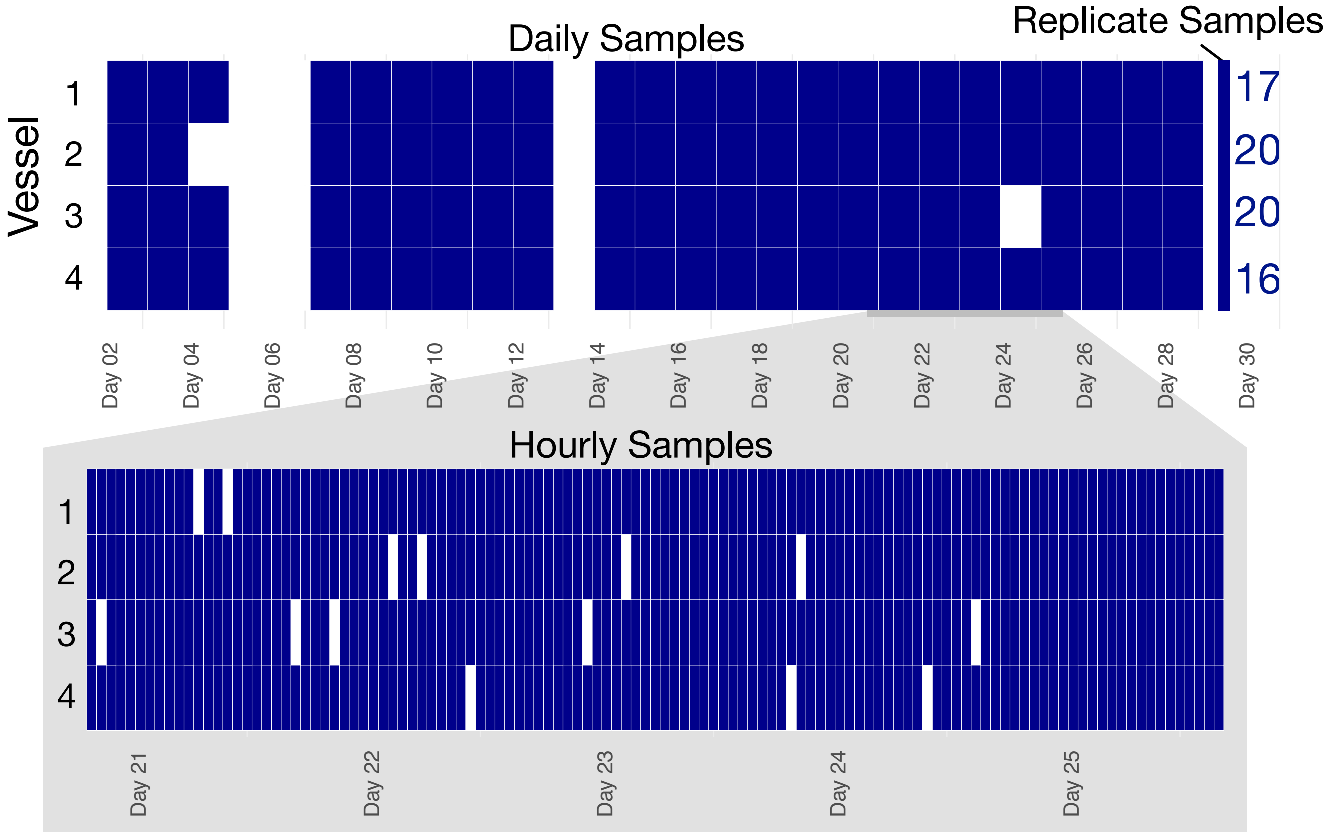

Daily Samples
Replicate Samples
Vessel
1
2
3
4
17
20
20
16
Day 02
Day 04
Day 06
Day 08
Day 10
Day 12
Day 14
Day 16
Day 18
Day 20
Day 22
Day 24
Day 26
Day 28
Day 30
Hourly Samples
1
2
3
4
Day 21
Day 22
Day 23
Day 24
Day 25
