# Supplementary material for: Dynamic linear models guide design and analysis of microbiota studies within artificial human guts
Source: Microbiome. 2018 Nov 12;6:202. doi: 10.1186/s40168-018-0584-3 (PMC6233358; doi:10.1186/s40168-018-0584-3)
Supplement: Supplementary file 15 — Dynamics inferred in the balance between the Enterobacteriaceae and all other taxa does not correlate with known external factors. As in Fig. 5d, the posterior 95% credible interval of the microbial dynamics (θ) for the balance between the Enterobacteriaceae and all other taxa shown. The posterior mean is colored according to the ID of the researcher who obtained each corresponding sample. Samples that were dropped from analysis due to low sequencing depth are denoted by NA for researcher ID. Time-points corresponding to the daily sampling regimen are shown in black. (PDF 42 kb) [file 40168_2018_584_MOESM15_ESM.pdf]

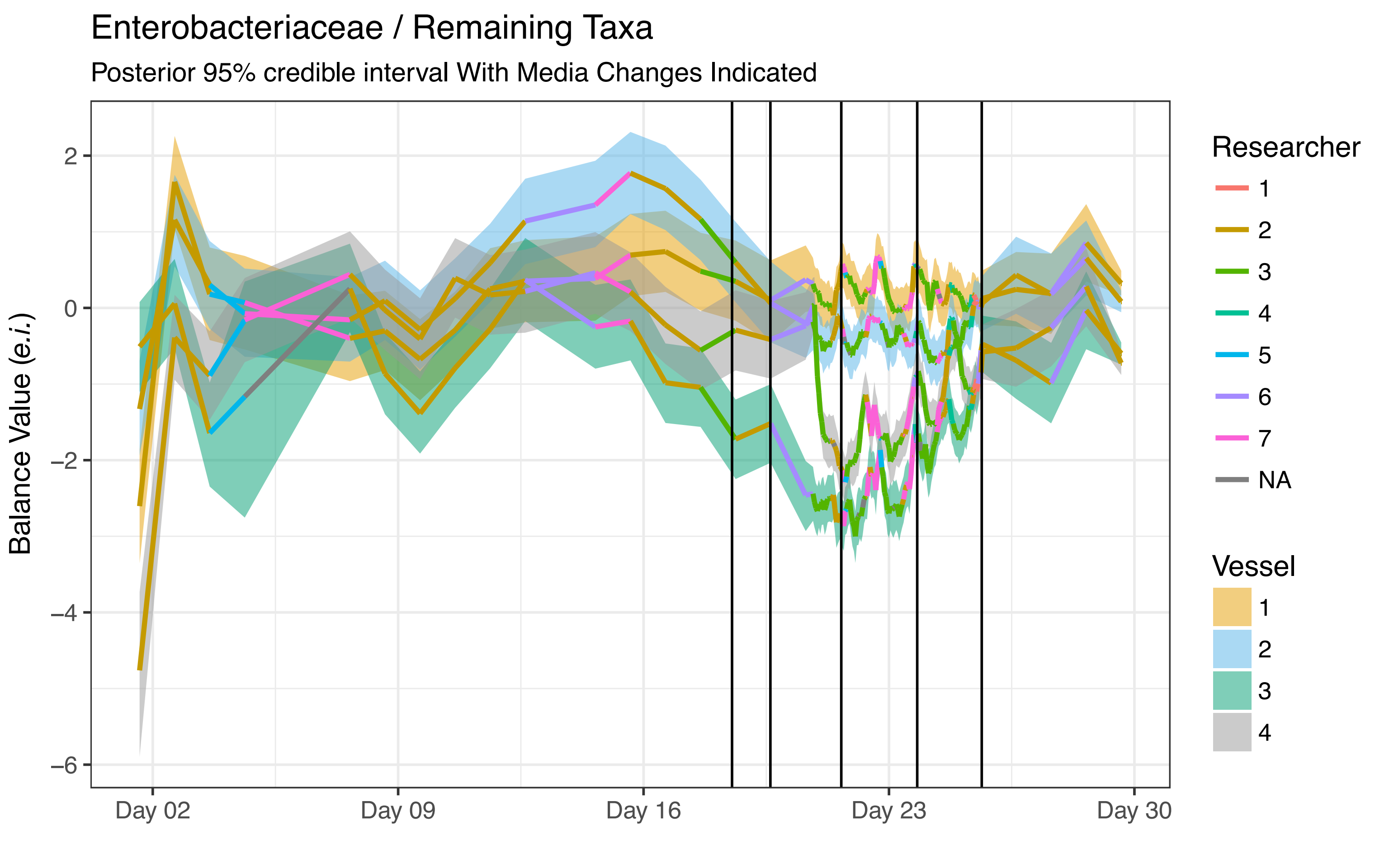

Enterobacteriaceae / Remaining Taxa
Posterior 95% credible interval With Media Changes Indicated
Balance Value (e.i.)
2
0
−2
−4
−6
Day 02
Day 09
Day 16
Day 23
Day 30
Researcher
1
2
3
4
5
6
7
NA
Vessel
1
2
3
4
