# Supplementary material for: Dynamic linear models guide design and analysis of microbiota studies within artificial human guts
Source: Microbiome. 2018 Nov 12;6:202. doi: 10.1186/s40168-018-0584-3 (PMC6233358; doi:10.1186/s40168-018-0584-3)
Supplement: Supplementary file 16 — Summary of the Kalman Filter, Kalman Smoother, and Backwards Sampling algorithm. (PDF 295 kb) [file 40168_2018_584_MOESM16_ESM.pdf]

Dynamic linear models guide design and analysis of microbiota studies within artificial human guts: Additional File

Justin D Silverman[1,2,3], Heather K Durand[5], Rachael J Bloom[4], Sayan Mukherjee[1,6], and Lawrence A David[1,3,4,5]

[1]Program in Computational Biology and Bioinformatics, Duke University, Durham, NC 27708
[2]Medical Scientist Training Program, Duke University, Durham, NC 27708
[3]Center for Genomic and Computational Biology, Duke University, Durham, NC 27708
[4]University Program in Genetics and Genomics, Duke University, Durham, NC 27708
[5]Department of Molecular Genetics and Microbiology, Duke University, Durham, NC 27708
[6]Departments of Statistical Science, Mathematics, Computer Science, Biostatistics & Bioinformatics, Duke University, Durham, NC 27708

Kalman Filter and Smoother

Our goal here is to demonstrate how the Kalman filter and smoother provide a means of calculating $p(\eta|\Xi) = p(\eta_1|\Xi)\prod_{k=2}^{K} p(\eta_k|\eta_{k-1},\dots,\eta_1,\Xi)$ and sampling $p(\theta|\eta, V, W, D, B)$[1]. Based on this goal, we can restrict our attention to a portion of the full MALLARD framework, particularly the conditional model

$$\begin{aligned}\eta_k &\sim N(F_k'\theta_k, V_k)\\ \theta_k &\sim N(G_k\theta_{k-1}, W_k)\\ \theta_0 &\sim N(m_0, C_0)\end{aligned}$$

where we consider η_k, V_k, W_k, F_k, and G_k as given for all $k \in \{1,\dots,K\}$. This presentation of the Kalman Filter and Smoother directly parallels the discussion presented by West and Harrison [1].

Theorem 1. ***(Kalman Filter)*** *For the above model, one-step forecast distributions of the form $p(\eta_k|\eta_{k-1},\dots,\eta_1,\Xi)$ and posterior distributions of the form $p(\theta_k|\eta_k,\dots,\eta_1,\Xi)$ are calculable for all k as follows:*

(a) Posterior at step $k-1$:
For some mean m_{k-1} and variance matrix C_{k-1},

$$p(\theta_{k-1}|\eta_{k-1},\dots,\eta_1,\Xi) \sim N(m_{k-1}, C_{k-1}).$$

(b) Prior at step k:

$$\begin{aligned}a_k &= G_k m_{k-1}\\ R_k &= G_k C_{k-1} G_k' + W_k\\ p(\theta_k|\eta_k,\dots,\eta_1,\Xi) &\sim N(a_k, R_k)\end{aligned}$$

(c) One-step forecast at step k:

$$\begin{aligned}f_k &= F' a_k\\ Q_k &= F_k' R_k F_k + V_k\\ p(\eta_k|\eta_{k-1},\dots,\eta_1,\Xi) &\sim N(f_k, Q_k) \qquad (1)\end{aligned}$$

[1] Just as in the main text we consider $\Xi = \{V, W, m_0, C_0, F, G\}$.

(d) Posterior at step k:

$$
\begin{aligned}
e_k &= \eta_k - f_k \\
A_k &= R_k F_k Q_k^{-1} \\
m_k &= a_k + A_k e_k \\
C_k &= R_k - A_k Q_k A_K' \\
p(\theta_k|\eta_k, \ldots, \eta_1, \Xi) &\sim N(m_k, C_k).
\end{aligned}
$$

Proof. The proof follows directly from the corresponding theorem given in West and Harrison [1] with the following substitutions: $t \leftarrow k$ throughout and $Y_k \leftarrow \eta_t$. □

Theorem 2. ***(Kalman Smoother)*** *The marginal posterior distribution for $p(\theta_k|\eta_K, \ldots, \eta_1, \Xi)$ can be sampled directly given quantities R_k, C_k, a_k, and m_k calculated from the Kalman filter as follows:*

(1) For step K:

$$
\begin{aligned}
h_K &= m_K \\
H_K &= C_K \\
p(\theta_K|\eta_K, \ldots, \eta_1, \Xi) &\sim (h_K, H_K)
\end{aligned}
$$

(2) For step $0 \leq k < K$:

$$
\begin{aligned}
B_k &= C_k G_{k+1}' R_{k+1}^{-1} \\
h_k &= m_k + B_k(h_{k+1} - a_{k+1}) \\
H_k &= C_k + B_k(H_{k+1} - R_{k+1})B_k' \\
p(\theta_k|\eta_K, \ldots, \eta_1, \Xi) &\sim (h_k, H_k)
\end{aligned}
$$

Proof. The proof follows directly from the discussion given in West and Harrison [1] with the substitution $t \leftarrow k$. □

The Kalman Smoother can be seen as a method for calculating the posterior marginals of θ_k. Sampling from these marginals is straight forward and reduces to sampling sequentially from the multivariate normal as demonstrated in Theorem 2. For many purposes, as was the case in the analysis of the artificial gut dataset, sampling from these marginals will be sufficient for posterior analysis. However, if sampling of entire trajectories from the joint posterior $p(\theta_K, \ldots, \theta_1|\eta_K, \ldots, \eta_1, \Xi)$ is desired, a different algorithm is required. While this task is in general more complicated than sampling from the posterior marginals, we present the Backwards Sampling Algorithm which solves this task. Our discussion of the Backwards Sampling algorithm will follow the presentation of West and Harrison [1].

(Backwards Sampling Algorithm) The posterior distribution for $p(\theta_K, \ldots, \theta_1|\eta_K, \ldots, \eta_1, \Xi)$ can be sampled from directly given quantities R_k, C_k, a_k, and m_k calculated from the Kalman filter as follows:

(1) At step K:

$$
\begin{aligned}
h_K &= m_K \\
H_K &= C_K \\
p(\theta_K|\eta_K, \ldots, \eta_1, \Xi) &\sim N(h_K, H_K)
\end{aligned}
$$

(2) For step $0 \leq k < K$:

$$
\begin{aligned}
B_k &= C_k G_{k+1}' R_{k+1}^{-1} \\
h_k &= m_k + B_k(\theta_{k+1} - a_{k+1}) \\
H_k &= C_k - B_k R_{k+1} B_k' \\
p(\theta_k|\theta_{k+1}, \eta_K, \ldots, \eta_1, \Xi) &\sim N(h_k, H_k)
\end{aligned}
$$

While the Algorithm is technically correct, care should be taken as certain models can cause the above algorithm to fail in practice due to singularity in the matrix H_k. For example, in analyzing two samples (k and $k+1$) that are technical replicates ($W_{k+1} = \mathbf{0}$), we will find that $R_{k+1} = G_{k+1}C_kG'_{k+1} + \mathbf{0}$ which will cause $B_k = C_kG'_{k+1}(G_{k+1}C_kG'_{k+1})^{-1}$ and therefore causing

$$\begin{aligned}
H_k &= C_k - C_kG'_{k+1}(G_{k+1}C_kG'_{k+1})^{-1}G_{k+1}C_kG'_{k+1}(G_{k+1}C_kG'_{k+1})^{-\prime}G_{k+1}C_k \\
&= C_k - C_kG'_{k+1}(G_{k+1}C_kG'_{k+1})^{-\prime}G_{k+1}C_k \\
&= C_k - C_kG'_{k+1}(G^{-\prime}_{k+1}C_k^{-1}G^{-1}_{k+1})G_{k+1}C_k \\
&= C_k - C_kC_k^{-1}C_k \\
&= \mathbf{0}.
\end{aligned}$$

To summarize, having $W_{k+1} = \mathbf{0}$ (as would be seen in the case of technical replicates, can cause $H_k = \mathbf{0}$ and therefore causing $p(\theta_k|\theta_{k+1}, \eta_K, \ldots, \eta_1, \Xi)$ to be singular and likely causing errors for most computer programs. However, this may be improved by adding a condition into the sampling that if $W_{k+1} = \mathbf{0}$ then θ_k may be calculated (not sampled) as $\theta_k = h_k$.

Observations Missing at Random

Observations missing at random (e.g., Y_k is missing at random) are often trivially handled by MALLARD using the ability of the Kalman filter to handle missing values. In particular, if observation k is missing, the fourth step of the Kalman filter (in which the Posterior at step k is computed) can be replaced with the following update rule:

$$\begin{aligned}
m_k &= a_k \\
C_k &= R_k \\
p(\theta_k|\eta_k, \ldots, \eta_1, \Xi) &\sim N(m_k, C_k).
\end{aligned}$$

In other words, in the presence of a missing observation the prior becomes the posterior. Importantly, this simple change to the Kalman filter is usually the only change required to allow MALLARD models to handle observations missing at random.

References

[1] Mike West, Jeff Harrison. *Bayesian Forecasting and Dynamic Models*. 2nd edn. New York: Springer; 1997. pgs. 116-120 and 582-584
