# Supplementary material for: Dynamic linear models guide design and analysis of microbiota studies within artificial human guts
Source: Microbiome. 2018 Nov 12;6:202. doi: 10.1186/s40168-018-0584-3 (PMC6233358; doi:10.1186/s40168-018-0584-3)
Supplement: Supplementary file 17 — Table of parameters in MALLARD model used to analyze artificial gut dataset. For the artificial gut dataset: R = 4, the number of artificial gut vessels; D = 10, the number of bacterial families analyzed; T(1) = 158, the number of sample points at which η is to be inferred; and T(2) = 138, the number of time-points at which θ is to be inferred. T(1) and T(2) differ due to technical replicates and time-points which lacked measurement but where inference of θ was still desired. Only the parameters η, ΛV, ΛW, σV, and σW are sampled using HMCMC. The parameters θ are sampled directly from the posterior using the Kalman smoother. (PDF 22 kb) [file 40168_2018_584_MOESM17_ESM.pdf]

Variable	Number of Parameters
η	5688 $T^{(1)} \times R \times (D-1)$
Λ^V	45 $(D^2 + D + 2)/2$
Λ^W	45 $(D^2 + D + 2)/2$
σ^V	9 $(D-1)$
σ^W	9 $(D-1)$
θ	4968 $T^{(2)} \times R \times (D-1)$
